# Trafficking and localization of Golgi-resident *N*-glycan processing enzymes in plants

**DOI:** 10.3389/fpls.2025.1624949

**Published:** 2025-07-25

**Authors:** Kai Dünser, Jennifer Schoberer

**Affiliations:** Department of Biotechnology and Food Science, Institute of Plant Biotechnology and Cell Biology, BOKU University, Vienna, Austria

**Keywords:** plants, Golgi apparatus, glycosylation, *N*-glycan processing, *N*-glycan processing enzymes, protein transport, glycoengineering

## Abstract

Asparagine (*N*)-linked glycosylation is a fundamental co- and post-translational modification of proteins, playing a crucial role in protein folding, stability and function, protein-protein interactions, biotic and abiotic stress response as well as glycan-dependent quality control processes in the endoplasmic reticulum (ER). Protein *N*-glycosylation is initiated in the ER and continued in the Golgi apparatus by *N*-glycan-processing glycosyltransferases and glycosidases, which are compartmentalized in a highly organized manner reflecting their function in the sequential modification of glycans. Therefore, the precise localization of these enzymes is crucial for the optimal functioning of the glycosylation process and the secretory pathway and hence must be tightly regulated to maintain protein function, cellular health, and overall organismal development. Here, we highlight recent developments that contribute to a better understanding of the localization mechanisms of this important class of Golgi residents and discuss future directions to move the field forward.

## Introduction

**Figure 1:**
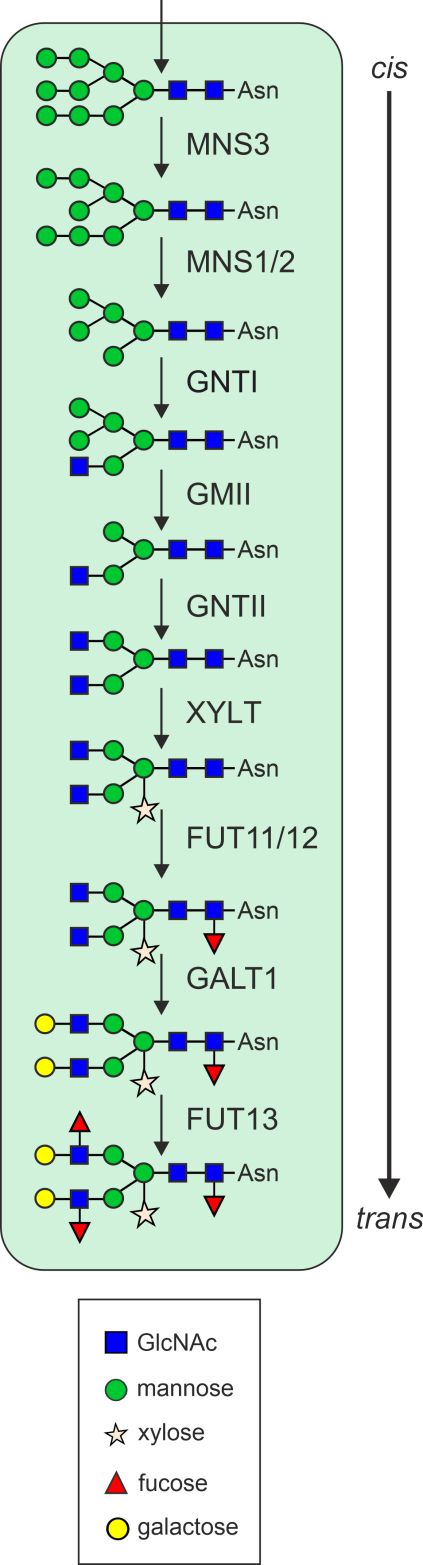
Schematic overview of the *N*-glycan processing steps in the plant Golgi apparatus. MNS3, ER-α-mannosidase I; MNS1/2, Golgi-α-mannosidase I; GNTI, β1,2-N-acetylglucosaminyltransferase I; GMII, Golgi-α-mannosidase II; GNTII, β1,2-*N*-acetylglucosaminyltransferase II; XYLT, β1,2-xylosyltransferase; FUT11/12, core α1,3-fucosyltransferase; GALT1, β1,3-galactosyltransferase; FUT13, α1,4-fucosyltransferase.

The *N*-glycosylation pathway begins in the ER, where the oligosaccharyltransferase complex catalyzes the *en bloc* transfer of a pre-assembled oligosaccharide precursor (Glc_3_Man_9_GlcNAc_2_) from the lipid carrier dolichol pyrophosphate to asparagine (Asn or *N*) residues within the specific consensus sequence Asn-X-Ser/Thr (X can be any amino acid except proline) of nascent proteins (Helenius and Aebi, 2001; Moremen et al., 2012; Strasser, 2016). Subsequently, the two outmost glucose residues are cleaved by α-glucosidase (GCS) I and GCSII, resulting in the generation of a monoglucosylated *N*-glycan. This glycan can interact with the lectins calnexin (CNX) and calreticulin (CRT), thereby promoting folding. The release from the CNX/CRT interaction depends on the trimming of the remaining glucose by GCSII. Proteins that fail to attain their final conformation are subjected to ER-associated degradation (ERAD), a process that involves the trimming of a specific mannose residue by the class I α-mannosidases MNS4 and MNS5 (Hüttner et al., 2014). This processing step produces a glycan degradation signal on the ERAD substrate, which is recognized by the lectin OS9 (Clerc et al., 2009; Quan et al., 2008; Strasser, 2018). Proteins that fold correctly, carry the Man_9_GlcNAc_2_ oligosaccharide, which is subsequently subjected to further processing by the class I α-mannosidases ER-α-mannosidase I (MNS3) and Golgi-α-mannosidase I (MNS1/2; Liebminger et al., 2009) in the Golgi apparatus (**Figure 1**). This collective action results in the removal of four α1,2-linked mannose residues from the *N*-glycan. The resulting Man_5_GlcNAc_2_ *N*-glycan is the final product of early *N*-glycan processing steps and serves as the acceptor substrate for *N*-acetylglucosaminyltransferase I (GNTI), which adds a single *N*-acetylglucosamine (GlcNAc) residue, thereby initiating the formation of hybrid and complex *N*-glycans in the Golgi apparatus (Strasser et al., 1999). The product of this reaction is further processed by Golgi-α-mannosidase II (GMII), *N*-acetylglucosaminyltransferase II (GNTII), β1,2-xylosyltransferase (XYLT) and core α1,3-fucosyltransferase (FUT11/12; Strasser et al., 2006). The final, mature *N*-glycans are of the complex type and are typically characterized by the presence of β1,2-xylose and α1,3-fucose (GnGnXF), which is considered to be the predominant plant glycoform (Zeng et al., 2018; Strasser et al., 2021). Variations thereof may lack terminal GlcNAc on one of the two branches (GnMXF, MGnXF) and additionally lack xylose and/or fucose (GnGnF, GnGnX, GnMX, MGnX, GnMF, MGnF, GnGn, GnM, MGn). Complex *N*-glycans can be converted into paucimannosidic *N*-glycans, which lack the two terminal GlcNAc residues (MM, MMF, MMX or MMXF) in plants. This processing step occurs in post-Golgi compartments and is carried out by either the vacuolar β-*N*-acetylhexosaminidase 1 (HEXO1) or HEXO3, which mainly resides in the plasma membrane/apoplast (Strasser et al., 2007a; Liebminger et al., 2011; Alvisi et al., 2021). The most elaborate occurring glycoform in plants is the Lewis A structure, which is generated in the *trans*-Golgi by β1,3-galactosyltransferase (GALT1) and α1,4-fucosyltransferase (FUT13) via the modification of GnGnXF structures (Strasser et al., 2007b). Lewis A *N*-glycans are ubiquitously found in plants (Fitchette et al., 1999; Wilson et al., 2001), but are only present on a few glycoproteins (Beihammer et al., 2021).

While the initial steps of *N*-glycosylation in the ER are highly conserved among eukaryotes, the processing steps in the Golgi show distinct differences between plants and mammals (Schoberer and Strasser, 2018). The presence of β1,2-xylose and α1,3-fucose is a defining feature of plant *N*-glycosylation, as these sugars are absent in mammalian systems (Strasser, 2016). This structural difference may contribute to the antigenicity of some plant proteins and increase the risk of adverse reactions, such as allergic reactions or immunological responses, when plant-produced recombinant glycoproteins with β1,2-xylose and α1,3-fucose are used in mammals in a therapeutic context (Jenkins et al., 1996; Bardor et al., 2003; Shaaltiel and Tekoah, 2016).

Our understanding of the role of *N*-glycans in plants is primarily derived from the study of mutants deficient in *N*-glycan biosynthesis and processing. These studies have demonstrated that defects in *N*-glycosylation can result in a range of adverse effects, including abnormal plant development and/or reduced stress tolerance (Kang et al., 2008; Fanata et al., 2013; Pedersen et al., 2017; Kaulfürst-Soboll et al., 2021). Mutations in genes encoding enzymes involved in the biosynthesis and early processing of *N*-glycans are embryo lethal (Strasser, 2014), whereas mutants of enzymes involved in *N*-glycan processing in the Golgi do not show any significant morphological phenotypes under normal growth conditions but may have conditional phenotypes (Strasser, 2022). This shows that the functions of *N*-glycans in the ER are essential, whereas Golgi-derived complex *N*-glycans are dispensable for plant survival but may have additional roles under non-physiological conditions.

## The plant Golgi apparatus and its role in *N*-glycan processing

**Figure 2:**
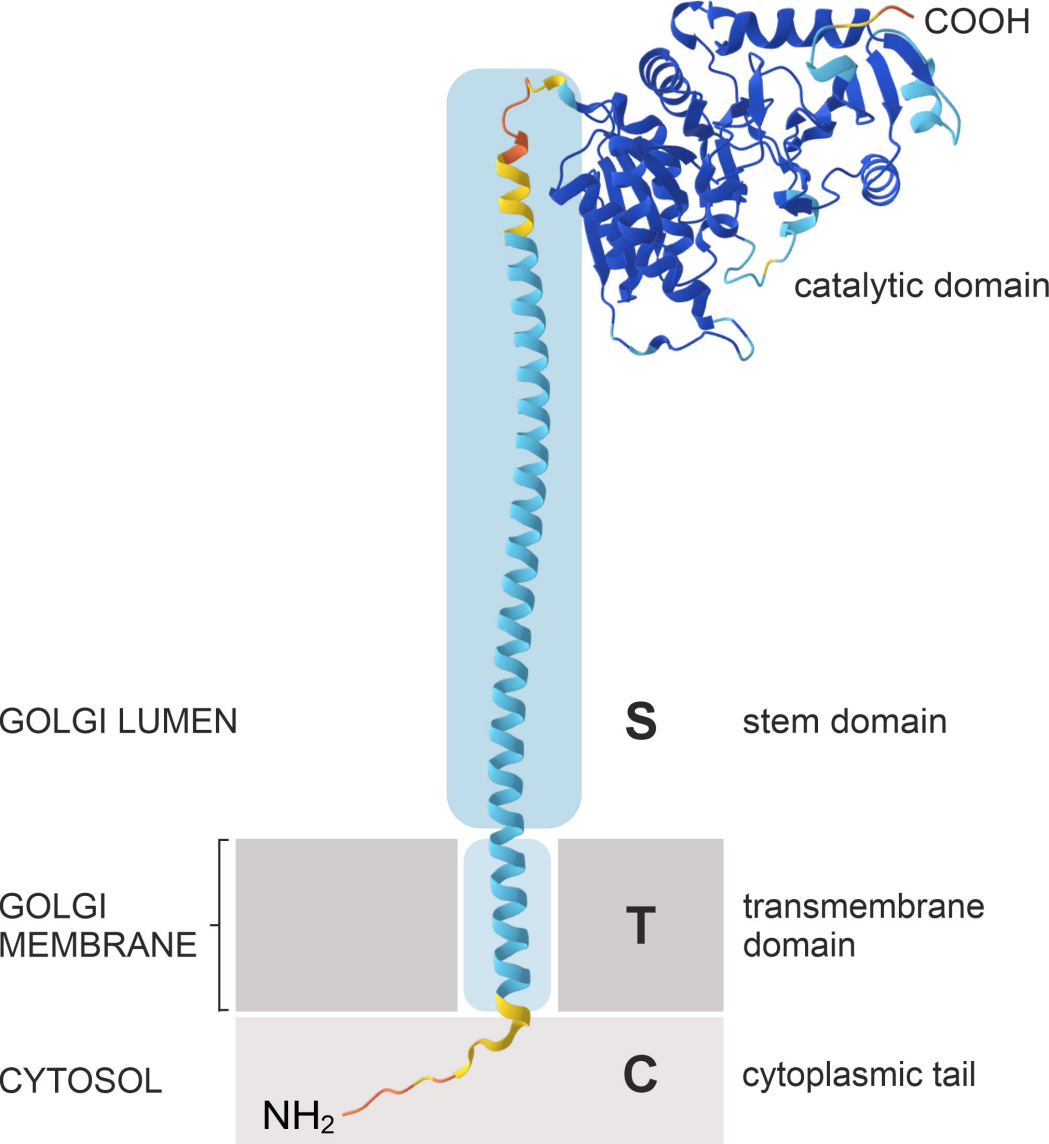
Schematic illustration of the predicted protein structure and domain organization of *N. tabacum* GNTI. NtGNTI is used as a representative model of plant Golgi *N*-glycan processing enzymes, which have a type II membrane topology, with the N-terminus (NH_2_) residing in the cytosol. This model is based on AlphaFold and DeepTMHMM (Hallgren et al., 2022) protein structure predictions.

The Golgi apparatus is the central biosynthetic organelle in the secretory pathway and the main site for the modification and sorting of proteins and lipids, and the biosynthesis of cell wall polysaccharides in plants (Foresti and Denecke, 2008). The plant Golgi apparatus has a unique morphology as it comprises numerous discrete stacks of flattened membrane compartments, classified into *cis*-, medial- and *trans*-Golgi cisternae. Plant Golgi stacks display an actin-myosin-driven mobility, distributing them throughout the cytoplasm in higher plants (Faso et al., 2009; Hawes et al., 2010). Golgi cisternae compartmentalize specific populations of resident proteins that catalyze the step-wise processing of *N*-glycans (Hawes and Satiat-Jeunemaitre, 2005; Schoberer and Strasser, 2011) or the assembly of cell wall polysaccharides (Moore et al., 1991; Oikawa et al., 2013). Golgi-localized *N*-glycan processing enzymes are type II transmembrane proteins comprising an N-terminal cytoplasmic tail (C), a single transmembrane domain (T) and a luminal stem region (S), collectively referred to as the CTS domain, which is linked to the large catalytic domain in the Golgi lumen (**Figure 2**). The localization of these enzymes within the Golgi stack is highly organized and closely linked to their *in vivo* substrate specificities. Each enzyme produces the substrate for the next, thereby forming an assembly line that enables the sequential modification of glycoproteins (**Figure 1**). Consequently, an enzyme’s specific role in the pathway is reflected by its enrichment in distinct regions of the Golgi stack, rather than by its precise confinement to a single cisterna (Saint-Jore-Dupas et al., 2006; Staehelin and Kang, 2008; Schoberer and Strasser, 2011). This results in the formation of a biochemical gradient across the stack from *cis* to *trans*, with enzymes that act early in the *N*-glycan processing pathway residing in *cis*- and medial-Golgi cisternae, whereas later-acting enzymes concentrate in medial- and *trans*-Golgi cisternae or the *trans*-Golgi network (TGN). This non-uniform, subcompartment-specific distribution pattern of Golgi-localised glycosidases and glycosyltransferases remains constrained despite the constant flux of cargo molecules and the constant mobility of the Golgi apparatus (Boevink et al., 1998) and provides a valuable tool for studying various aspects of Golgi (protein) organization, trafficking and biogenesis (Hawes et al., 2010; Schoberer et al., 2010).

**Figure 3:**
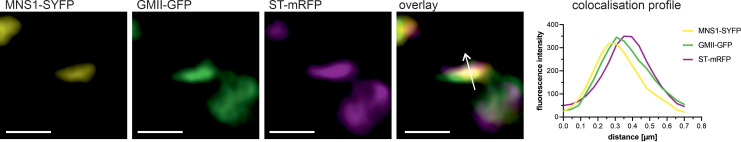
Co-localization of fluorescent protein-labelled *N*-glycan processing enzymes within the Golgi. Confocal microscopy images show a single, highly magnified Golgi stack triple-labelled with three different *N*-glycan processing enzymes with distinct intra-Golgi distributions. The fusion proteins MNS1-SYFP (CTS region fused to yellow fluorescent protein, in yellow), GMII-GFP (CTS region fused to green fluorescent protein, in green), and ST-mRFP (CTS region fused to red fluorescent protein, in magenta) were transiently co-expressed in *N. benthamiana* leaf epidermal cells and imaged using a Zeiss LSM980 Airyscan 2 confocal microscope. Images were further processed with the ZEN module Airyscan joint deconvolution. The co-localization profile shows the fluorescence intensities of the fusion proteins, plotted along the white arrow in the overlay image. Note the shifts in the fluorescence intensity peaks, indicating an enrichment of enzymes in distinct Golgi regions. Scale bar = 1 µm.

The initial evidence supporting the subcompartmentation of glycosidases and glycosyltransferases within the plant Golgi apparatus was based on the localization of the polysaccharide and glycoprotein products rather than the enzymes themselves (Moore et al., 1991; Lynch and Staehelin, 1992; Zhang and Staehelin, 1992). This was due to the absence of cloned plant Golgi-resident enzymes at that time. The cloning of the genes encoding plant glycosylation enzymes, coupled with the advent of fluorescent protein technology and confocal microscopy, has allowed the definitive determination of their subcellular localization within plant Golgi stacks (Essl et al., 1999; Dirnberger et al., 2002; Strasser et al., 2005; Saint-Jore-Dupas et al., 2006; Strasser et al., 2007b; Liebminger et al., 2009; Schoberer et al., 2009). The results of several studies have indicated that the fluorescent signals emitted by distinct co-expressed *N*-glycan processing enzymes do not entirely overlap within Golgi stacks, thereby substantiating the prevailing notion of a non-uniform distribution of glycosylation enzymes (**Figure 3**). This observation prompted extensive research to identify the precise signals and mechanisms by which this non-uniform Golgi distribution is established, maintained and regulated. It became evident early on that in plants the information required for the Golgi targeting and retention of plant *N*-glycan processing enzymes is present in their CTS region. The expression of the fluorescent protein-tagged CTS region of all plant *N*-glycan processing enzymes studied so far showed that the catalytic domain is not required for their targeting to the Golgi as their Golgi localization remained unchanged compared to the full-length enzymes (Essl et al., 1999; Dirnberger et al., 2002; Saint-Jore-Dupas et al., 2006; Schoberer et al., 2009, 2010; Schoberer and Strasser, 2011). This is in agreement with mammalian and yeast studies that localised the necessary signals for Golgi targeting to the transmembrane domain (TMD) and neighboring polypeptide regions of the enzymes (Munro, 1991; Nilsson et al., 1991; Colley, 1997). Furthermore, a fluorescent protein fusion to the CTS region of the rat α2,6-sialyltransferase (ST) has become the most commonly used Golgi marker in plants, with the protein localised to the *trans*-half of Golgi stacks in leaves of *Nicotiana clevelandii* as well as in callus tissue and root tips of Arabidopsis as demonstrated by electron microscopy (EM) (Boevink et al., 1998; Wee et al., 1998; Reichardt et al., 2007). This localization is consistent with the fact that the sialylation of glycoproteins by ST is a late *N*-glycan modification event in the Golgi of mammalian cells and it suggests that, despite the known differences between the plant and mammalian Golgi apparatus, the Golgi targeting signals and mechanisms are largely conserved between plants and mammals. This has been supported by studies showing that the plant and mammalian GNTIs can complement each other (Gomez and Chrispeels, 1994; Bakker et al., 1999) and that Golgi-targeted mammalian glycosyltransferases such as ST are functional when transiently or stably expressed in plants (Castilho et al., 2010; Kallolimath et al., 2016).

## Models for protein traffic through the Golgi

**Figure 4:**
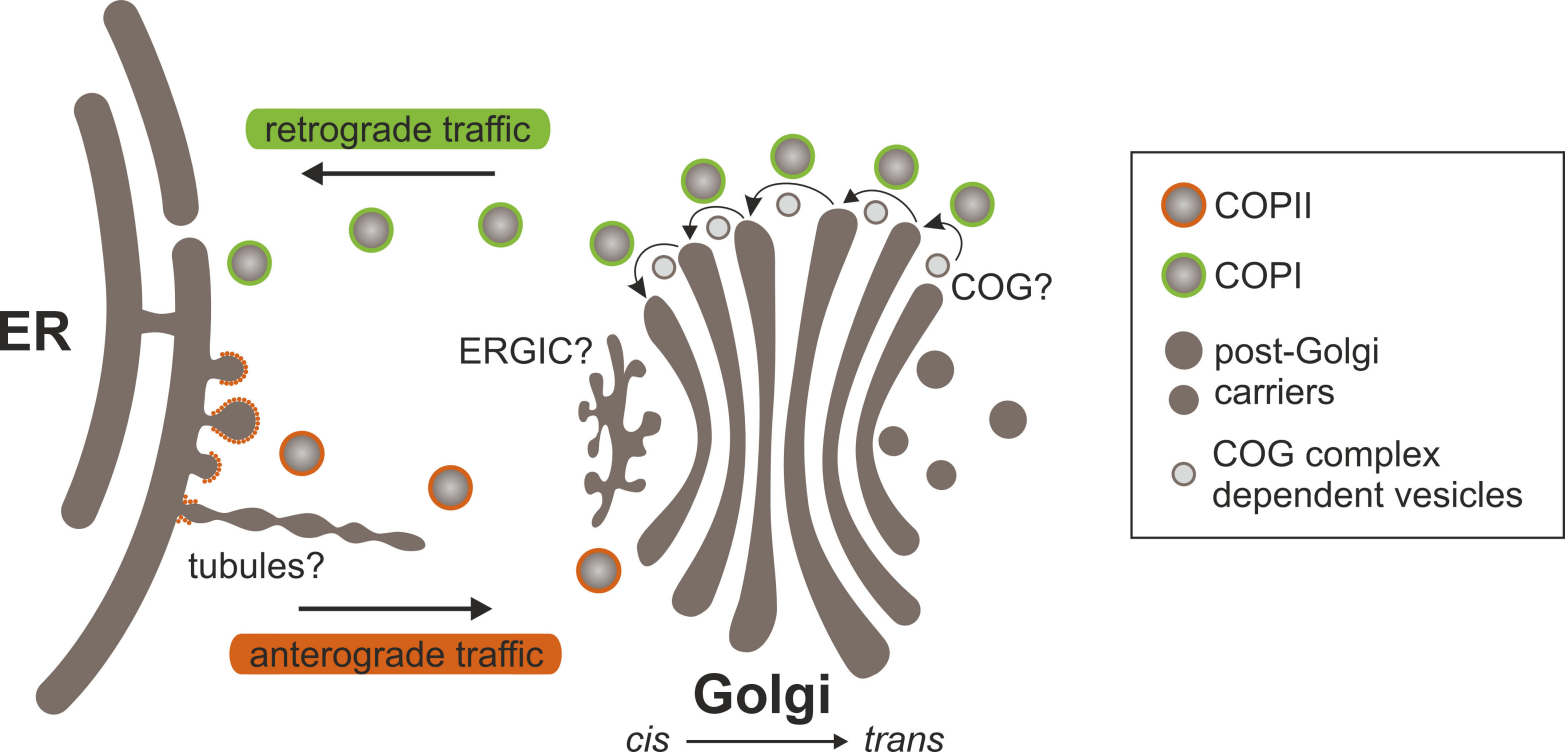
Protein transport between the ER and the Golgi. At ERES, cargo proteins are recruited and packaged into COPII transport carriers (vesicles and/or tubules) that bud from and exit the ER. COPII components can coat vesicles and/or decorate the neck of tubules. Carriers arrive at and fuse with *cis*-Golgi cisternae, releasing their contents. Cargo proteins move through the Golgi in a *cis*-to-*trans* direction in anterograde transport vesicles and/or via cisternal maturation. Anterograde transport probably involves elements of both models. Secretory proteins exit the Golgi at the *trans* face in post-Golgi transport carriers. Golgi residents are returned to earlier Golgi cisternae or the ER in COPI-coated vesicles that bud from Golgi cisternae. It is still unclear whether the COG complex plays a role in the retrograde intra-Golgi transport of Golgi-resident *N*-glycan processing enzymes. Another mystery is the functional role of the ERGIC in ER-Golgi transport. It may serve as an intermediate compartment where anterograde and retrograde transport pathways to and from the Golgi converge.

Several models have been proposed to elucidate the distinct localization of glycosylation enzymes within *cis*-, medial-, and *trans*-Golgi cisternae. These models seek to elucidate how enzymes are sorted, retained or recycled within different Golgi cisternae, thereby ensuring the optimal functioning of glycosylation processes. Nevertheless, models that address the sorting and concentration of glycosylation enzymes fundamentally depend on the characteristics of protein transport through the Golgi apparatus (Glick and Nakano, 2009). This highly regulated process can entail anterograde (forward) movement from *cis*- to *trans*-Golgi cisternae and retrograde (backward) movement between cisternae or from the Golgi to the ER (**Figure 4**). Several mechanisms have been put forth to explain the transport of glycosylation enzymes through the Golgi apparatus.

The vesicular transport model was influential in early mammalian cell biology and is based on the discovery “of machinery regulating vesicle traffic, a major transport system in our cells”, which led to the awarding of the 2013 Nobel Prize in Physiology or Medicine to James Rothman, Randy Schekman and Thomas Südhof (Zierath and Lendahl, 2013). It suggests that Golgi cisternae are stable compartments that house an unchanging set of Golgi-resident proteins (including glycosylation enzymes). Anterograde transport vesicles that form in the Golgi arrive at each cisterna with cargo proteins that are modified by resident enzymes within the cisterna. New vesicles with cargo proteins bud from the cisterna and then travel to the next stable cisterna in a *cis* to *trans* direction, where the next set of enzymes further modifies the cargo (Balch et al., 1984; Rothman and Wieland, 1996; Farquhar and Palade, 1998).

The cisternal maturation model suggests that each Golgi cisterna matures over time from the *cis* to the *trans* face of the Golgi, rather than being static, reflecting the ongoing process of protein modification and sorting. Proteins move as passengers within cisternae through the Golgi stack (Losev et al., 2006; Matsuura-Tokita et al., 2006; Emr et al., 2009). In this model, a new *cis*-Golgi cisterna matures into a medial- and then *trans*-Golgi cisterna before breaking apart as modified proteins are packaged for transport to their final destination, such as the plasma membrane or secretion outside the cell. Glycosylation enzymes are retained in specific cisternae by selective retrieval processes; enzymes that are needed in the earlier stages of glycosylation, are recycled from the later Golgi compartments back to earlier, younger cisternae by retrograde transport carriers, rather than being transported from one cisterna to another, to maintain the proper enzyme composition (Glick et al., 1997; Pelham, 1998). Yeast provided the most direct evidence via real-time tracking of cisternal markers and cargo. Meanwhile, algal and mammalian studies highlighted the model’s applicability to diverse cargo types, including bulky proteins.

In the rapid partitioning model, the Golgi apparatus is considered a highly dynamic two-phase membrane system, in which secretory cargo proteins and resident glycosylation enzymes are continuously and rapidly partitioned into specific lipid domains of optimal composition, so-called “export domains”, which are enriched in cargo and “processing domains”, which are enriched in Golgi-resident enzymes (Patterson et al., 2008). Cargo moves forward by stochastically exiting from export domains at any cisternae. This model accounts for the high efficiency of protein sorting and processing in the Golgi while maintaining its compartmentalized structure; however, experimental evidence is currently limited to mammalian cells.

The cisternal progenitor model proposes that new cisternae are continuously formed at the *cis*-face as progenitors or “precursors” of mature cisternae (Pfeffer, 2010). These progenitor cisternae then gradually mature as they move through the Golgi stack from *cis* to *trans*, where they are eventually disassembled or recycled to form new cisternae. As the cisternae mature, they progressively acquire different enzymes that process cargo proteins in stages. RAB GTPases play a crucial regulatory role in this model by coordinating the formation, maturation, and function of Golgi cisternae as they move through the stack. RAB GTPases are a family of small GTP-binding proteins that act as molecular switches, switching between active (GTP-bound) and inactive (GDP-bound) states. Their primary role is to regulate vesicular trafficking including cargo sorting, vesicle budding, and fusion by recruiting specific effector proteins (Woollard and Moore, 2008; Stenmark, 2009). In the cisternal progenitor model, RABs are thought to act in a cascade in which one RAB controls the recruitment of the next RAB, orchestrating the sequential maturation of cisternae. As a cisterna matures from *cis* to *trans*, another set of RABs takes over to regulate this progression. This cascade helps maintain the spatial organization of the Golgi and ensures efficient processing and sorting of proteins. This model takes into account both the maturation of cisternae and the dynamic recycling or exchange of membrane material, and it attempts to explain how the Golgi can maintain its structure and function while continuously processing large amounts of protein. This model has experimental support primarily in yeast (Rivera-Molina and Novick, 2009).

In plants, no experimental evidence has yet been presented to support the cisternal progenitor or rapid partitioning models. Instead, experimental evidence collectively favors the cisternal maturation model. For example, the interference with the retrograde transport machinery leads to the incorrect localization of Golgi residents and defects in Golgi stack morphology, which is consistent with impaired cisternal maturation, as the proper recycling of resident proteins and maintenance of cisternal identity are disrupted. Furthermore, large cargo molecules, such as cell wall polysaccharides, were retained in the cisternae and not recycled (Hawes and Satiat-Jeunemaitre, 2005; Glick and Nakano, 2009; Hawes et al., 2010; Robinson, 2020; Rui et al., 2022).These findings suggest that cisternal maturation may be a universal and evolutionarily conserved mechanism across all eukaryotes.

## Models for Golgi enzyme localization

Irrespective of the mechanisms by which Golgi proteins are transported through the Golgi, several models have been proposed to explain how the distinct sub-Golgi localization of glycosylation enzymes is established (**Table 1**).

**Table 1:** Sorting signals of various ER- and Golgi-resident membrane proteins.

Role in localisation	Protein	Organism	Topology	Interaction partner	Reference
ER export via anterograde transport					
Arginine/lysine-based cytoplasmic tail motifs					
R/K	prolyl 4-hydroxylase	plants	type II		Yuasa et al., 2005
KK	bZIP28	plants	type II	SAR1b	Srivastava et al., 2012
R/K	GNTI, GMII, XYLT	plants	type II		Schoberer et al., 2009
(RK)X(RK)	GalT2, GalNAcT, SialT2	mammals	type II	SAR1	Giraudo and Maccioni, 2003; Quintero et al., 2010
Hydrophobic residues in the cytoplasmic tail					
FVXXXY	EMP12	plants	multi-TMD		Gao et al., 2012
FF	ERGIC-53	mammals	type I	SEC23/SEC24	Kappeler et al., 1997
YYXF	Emp47p, Emp46p	yeast	multi-TMD		Schröder et al., 1995; Sato and Nakano, 2002
YNNSN and LXXME	Sed5p	yeast	type II	SEC24	Mossessova et al., 2003
LXXLE plus dibasic R/K motif	BET12	plants	type II	SAR1	Chung et al., 2018
LXXLE	Bet1p	yeast	type II	SEC24	Mossessova et al., 2003
Diacidic cytoplasmic tail motifs					
(D/E)XE	GONST1	plants	multi-TMD		Hanton et al., 2005
	CASP	plants	type II		Hanton et al., 2005
	KAT1	plants	multi-TMD	SEC24A	Mikosch et al., 2006; Sieben et al., 2008
EXXD	SYP31	plants	type II		Chatre et al., 2009
ER retention via retrograde transport					
Dilysine-based cytoplasmic tail motifs					
KKXX	p24 family proteins	plants	type I	β'-COP, ARF1	Contreras et al., 2004; Langhans et al., 2008; Gao et al., 2014
KKXX	Cf-9	plants	type I		Benghezal et al., 2000
KXKXX	FAD3	plants	multi-TMD		McCartney et al., 2004
KK	GPAT8	plants	multi-TMD		Gidda et al., 2009
Arginine-based cytoplasmic tail motifs					
RR, RXR or RXXR (plus luminal domain)	GCSI	plants	type II		Boulaflous et al., 2008
RRXXXXR	M1-SAT-I (ST3GAL5)	mammals	type II		Uemura et al., 2009
Hydrophobic cytoplasmic tail motifs					
ϕ-X-X-K/R/D/E-ϕ	FAD2	plants	multi-TMD		McCartney et al., 2004
	GPAT9	plants	multi-TMD		Gidda et al., 2009
Golgi retention					
Cytoplasmic tail motifs					
LPYS	MNS3	plants	type II		Schoberer et al., 2019b
ϕ-(K/R)-X-L-X-(K/R)	GALNT3, GALNT8, C2GNT1	mammals	type II	β-, δ- and ζ-COP	Liu et al., 2018
	GALNT6	mammals	type II	β- and δ-COP	Liu et al., 2018
WX(n1-6)(W/F)	GALNT4	mammals	type II	β-, δ- and ζ-COP	Liu et al., 2018
Adaptor-binding via cytoplasmic tail					
(F/L)-(L/I/V)-X-X-(R/K)	*cis*- and medial Golgi mannosyltransferases, e.g.:				
	Kre2, Och1, Mnn9, Mnn2, Mnn5, Ktr6	yeast	type II	Vps74p	Tu et al., 2008, 2012
L-X-X-(R/K)	glycosphingolipid biosynthetic enzymes, O- and *N*-glycosylation enzymes, e.g.:				
	LCS, GD3S, GM3S, Gb3S	mammals	type II	GOLPH3	Rizzo et al., 2021
	POMGNT1, GALNT12	mammals	type II	GOLPH3	Pereira et al., 2014
	C1GALT1, GALNT2, GALNT7, GCNT1, MGAT1, MGAT2	mammals	type II	GOLPH3/GOLPH3L	Welch et al., 2021
	SiaT2 (ST6GAL1)	mammals	type II	GOLPH3/GOLPH3L	Eckert et al., 2014; Isaji et al., 2014; Welch et al., 2021
	C2GNTI (GCNT1)	mammals	type II	GOLPH3	Ali et al., 2012; Eckert et al., 2014
Via transmembrane domain					
glutamine (Q)	GNTI	plants	type II		Schoberer et al., 2014
cysteine (C) and histidine (H)	β1,4-GALT	mammals	type II		Aoki et al., 1992
length	ManI	plants	type II		Saint-Jore-Dupas et al., 2006
receptor interaction	ERManI	yeast	type II	Rer1p	Massaad et al., 1999
Via enzyme oligomerisation					
heteromerisation via stem	NAGTI	mammals	type II	MannII	Nilsson et al., 1994
homomerisation via stem	GNTI	plants	type II	GNTI	Schoberer et al., 2014, 2023
homomerisation via TMD	β1,4-GALT	mammals	type II	β1,4-GALT	Yamaguchi and Fukuda, 1995

ϕ are hydrophobic amino acid residues, X denotes any amino acid.

## Golgi retention via enzyme oligomerization

**Figure 5:**
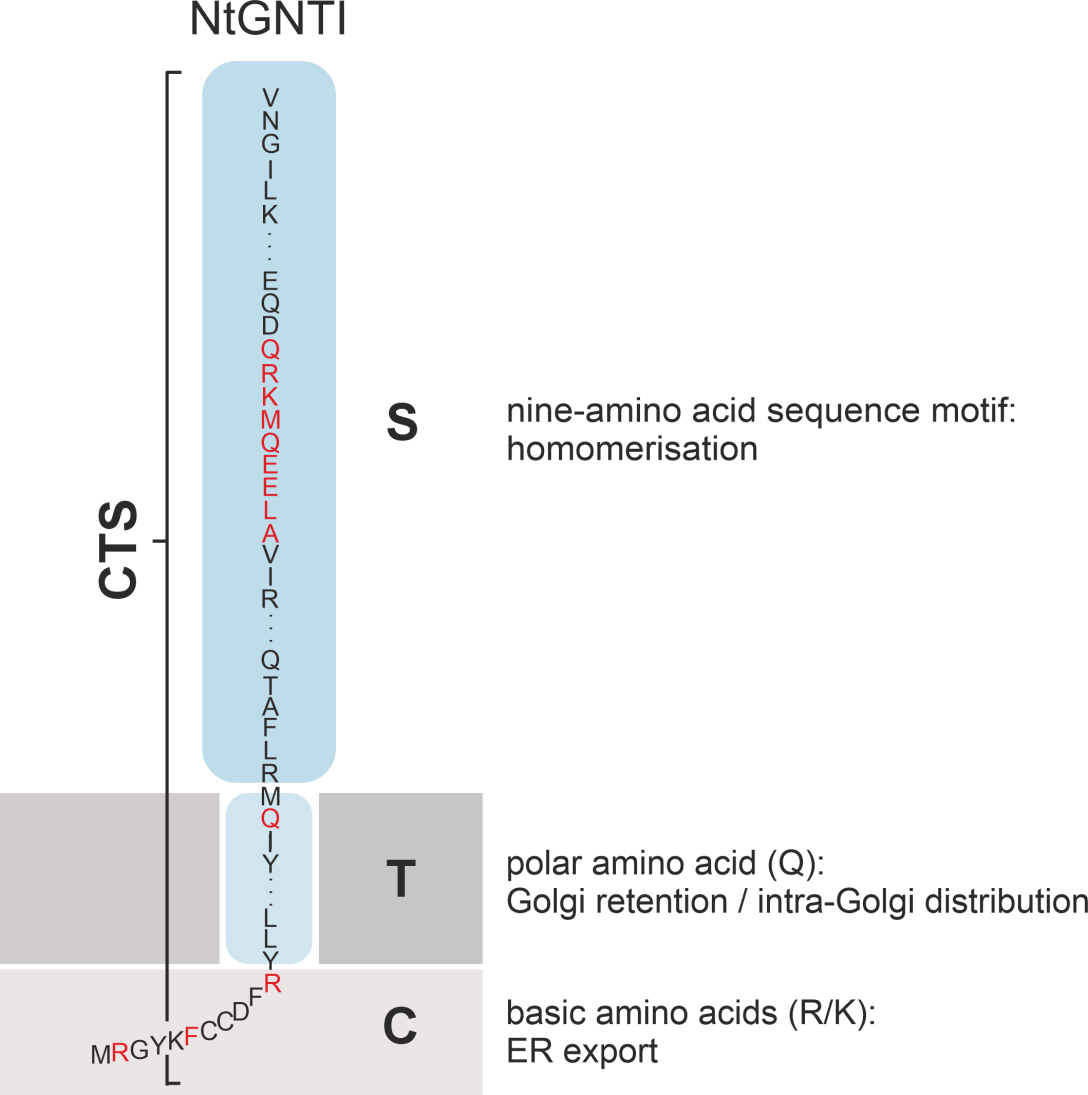
Schematic illustration showing the CTS region (cytoplasmic tail, transmembrane domain and stem domain) of NtGNTI. Amino acids and motifs in the different domains that are involved in the targeting and localization of NtGNTI to the Golgi are marked in red.

In the kin recognition model, Golgi enzymes form multimeric enzyme complexes that are unable to enter anterograde transport carriers due to their size, leading to the retention within specific Golgi cisternae (Nilsson et al., 1993). This complex formation may also allow for coordinated enzyme activity, ensuring that glycosylation occurs in the correct sequence as glycoproteins move through the Golgi. There is ample experimental evidence that the signals influencing oligomerization reside in the TMD and/or stem domain; however, the requirements for enzyme oligomerization vary greatly (Tu and Banfield, 2010). For example, the stem region of human NAGTI is the primary determinant for the heteromerization with mannosidase II (MannII), which is critical for their localization to the medial-Golgi in HeLa cells (Nilsson et al., 1994). The oligomerization of the sialyltransferase ST6Gal1, primarily via its TMD, is critical for its localization to the *trans*-Golgi (Chen and Colley, 2000). In plants, *N*-glycan processing enzymes such as MNS1, GNTI, GMII and XYLT have been shown to form *in planta* homo- and heterodimers in the *cis*- and medial-Golgi via their CTS region (Schoberer et al., 2013). Further biochemical approaches and confocal microscopy in *N. benthamiana* have demonstrated the role of the GNTI stem region in mediating homo- and heteromeric complex formation (Schoberer et al., 2014). A more recent study showed that a conserved nine-amino acid sequence motif in the stem was responsible for the GNTI-GNTI homodimerization, which led to a block in *N*-glycan processing increasing oligo-mannosidic *N*-glycans upon transient GNTI-CTS overexpression in *N. benthamiana*. Schoberer et al., 2023; **Figure 5**).

Of note, the formation of enzyme complexes is a common means of localizing enzymes involved in the biosynthesis of cell wall polysaccharides in the Golgi (Zabotina et al., 2021; **Table 1**). In contrast, homo- or heteromerization of *N*-glycan processing enzymes does not seem to be required for correct Golgi localization or catalytic activity.

## Golgi retention via transmembrane domains

**Figure 6:**
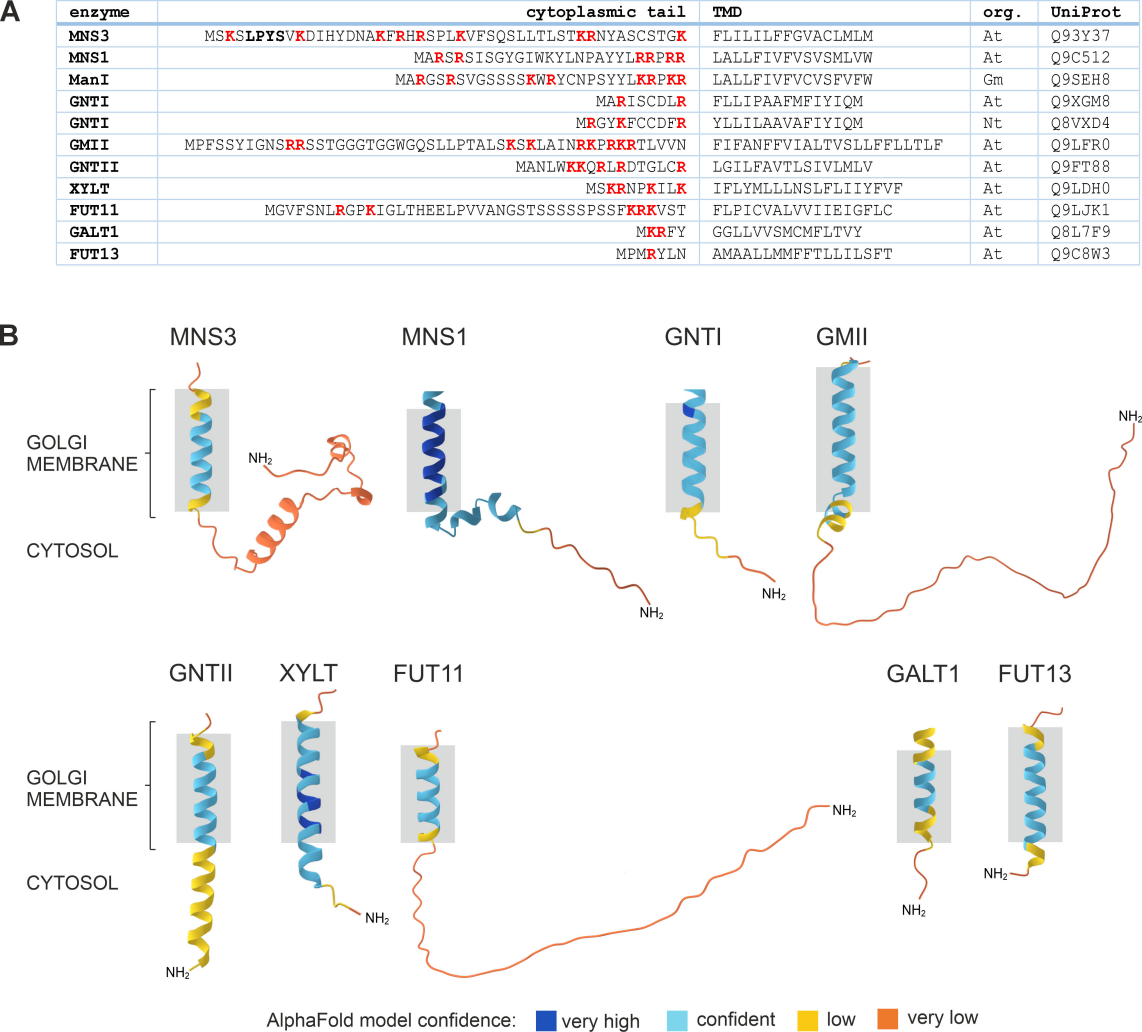
Comparison of the cytoplasmic tail and transmembrane domain (TMD) sequences of different plant Golgi-resident *N*-glycan processing enzymes. **(A)** Protein sequences were retrieved from the UniProt database. DeepTMHMM was used to predict the length and topology of the TMDs. Please note that TMD lengths are generally not determined experimentally and predictions are not always precise and vary depending on the used algorithm (Davis et al., 2006). Basic amino acids in the tail are shown in bold red. The LPYS Golgi retention motif of MNS3 is shown in bold. Org.: organism, At: *Arabidopsis thaliana*, Gm: *Glycine max*, Nt: *Nicotiana tabacum*. **(B)** Predicted 3D structures of the cytoplasmic tail and TMD regions of selected Golgi *N*-glycan processing enzymes from Arabidopsis (see panel **A**) as predicted by DeepTMHMM and AlphaFold. Grey boxes indicate TMD length and membrane anchoring.

The lipid bilayer model suggests that different membrane thicknesses within the Golgi stack naturally sort glycosylation enzymes into the appropriate locations based on their TMD length, preventing them from moving to the wrong compartment. The thickness of membranes increases progressively from the ER to the plasma membrane (PM). A mammalian study indicated that proteins localised in the ER and Golgi tend to have shorter TMDs than those in the PM, which suggests that shorter TMDs may result in retention in the Golgi apparatus, whereas longer TMDs facilitate progression to post-Golgi compartments and the PM (Welch and Munro, 2019). It has been demonstrated that the length of the TMD is a significant factor in the targeting of type I transmembrane proteins in plants (Brandizzi et al., 2002b). Immuno-EM studies demonstrated that an increase in TMD length from 16 to 23 residues resulted in the translocation of soybean α-mannosidase I (GmManI) from the *cis*- to the *trans*-Golgi (Saint-Jore-Dupas et al., 2006). Furthermore, the TMD of ER-localised GCSI would direct the protein to the Golgi, but for ER localization it requires ER retention signals, such as four arginine residues within its 13-amino-acids cytoplasmic N-terminus or the 60-amino-acids luminal C-terminus (Saint-Jore-Dupas et al., 2006; Boulaflous et al., 2008). However, this model does not consider that the TMD length of Golgi-resident enzymes varies considerably (**Figure 6A**). The *trans*-Golgi enzymes ST and GALT1, for example, have a rather short membrane-spanning region with a length of 17 and 15 amino acids, respectively, which is in a similar range to the predicted TMD lengths of the *cis*/medial-located enzymes MNS1 and GNTI, but much shorter than the TMD of GMII in the medial-Golgi (**Figure 6**; Saint-Jore-Dupas et al., 2006; Strasser et al., 2006; Liebminger et al., 2009; Schoberer et al., 2009; Schoberer and Strasser, 2011). A study investigating the TMDs of integral membrane proteins in fungi and mammals revealed organelle-specific variations in the length and composition of these TMDs (Sharpe et al., 2010). However, the analysis showed no significant differences in mean residue hydrophobicity or amino acid volume between *cis*- and medial-Golgi proteins. Also, the dataset used to examine specific characteristics of transmembrane segments was relatively limited, as only a restricted number of proteins with confirmed sub-Golgi localization were available.

Nevertheless, it is evident that while the TMD is of vital importance for the anchoring and sorting of glycosylation enzymes in the Golgi, correct localization is largely dependent on the presence of additional signals. To illustrate, the cytoplasmic tail of XYLT is indispensable for its Golgi localization, as its TMD alone targeted the enzyme to the PM, PM-derived vesicles and the ER (Dirnberger et al., 2002). Similarly, the TMD of GNTI alone showed cytoplasmic localization and partial colocalization with the GFP-tagged Golgi marker ER RETENTION DEFECTIVE 2 (ERD2). However, fusing the cytoplasmic tail of GNTI with the TMD restored its Golgi localization in *N. benthamiana* leaf cells (Schoberer et al., 2009). Furthermore, an N-terminal truncation of the cytoplasmic tail of GMII, comprising 10 amino acids most proximal to the TMD, was sufficient for its Golgi localization (Strasser et al., 2006). For the human GalNAc transferase family of mucin-type O-glycosylation enzymes, it was shown that a combination of the cytoplasmic tails and TMDs of GalNAc-T1, GalNAc-T2 and GalNAc-T7, respectively, and the combined TMD and luminal stem region of GalNAc-T7 and GalNAc-T10 resulted in Golgi localization (Becker et al., 2018).

Specific amino acids in the TMD have also been attributed a role in Golgi retention. For example, a conserved polar glutamine (Q) residue in the TMD of plant GNTI is crucial for maintaining its *cis*/medial-Golgi localization (Schoberer et al., 2019a; **Figure 5**). In contrast, the Golgi retention of human β1,4-galactosyltransferase relies on a cysteine and histidine residue within its TMD, which confer homodimerization (Aoki et al., 1992; Yamaguchi and Fukuda, 1995).

## ER export via anterograde transport

There is overwhelming evidence that the N-terminal cytoplasmic tail of *N*-glycosylation enzymes contains amino acid signal motifs that are recognized by the trafficking machinery (**Table 1**). The plant *N*-glycan processing enzymes GNTI, GMII and XYLT contain several arginine and/or lysine residues in their cytoplasmic tails, which are essential for ER export and Golgi localization (**Figures 5**, **6A**). A comparison of the cytoplasmic tails of plant *N*-glycan processing enzymes reveals a clear enrichment of basic, positively charged arginine and lysine residues, which frequently occur in clusters. Interestingly, protein structure predictions suggest that the length of the tails varies considerably and that the majority lack a defined secondary structure. This is most likely to facilitate the interaction with the sorting and transport machinery via motifs therein (**Figure 6B**). Evidence for COPII-dependent transport was provided when the mutagenesis of the three basic amino acids in the cytoplasmic tail of GNTI impaired the recruitment of tobacco SAR1 to ER export sites (ERES) (Schoberer et al., 2009, **Figure 5**). SAR1 is a small GTPase that initiates the formation of coat protein complex II (COPII) carriers by recruiting coat proteins (SEC23/24, SEC13/31) to the ER membrane to form the COPII coat. This coat helps to select cargo proteins for transport to the Golgi apparatus. Upon hydrolysis of GTP to GDP, SAR1 triggers the disassembly of the COPII coat, allowing the carrier to fuse with the Golgi. In mammals, the conserved dibasic amino acid motif (R/K)X(R/K) in the N-terminal cytoplasmic tail of mammalian glycosyltransferases involved in glycolipid synthesis has been shown to bind directly to SAR1 (Giraudo and Maccioni, 2003). The same group demonstrated by cytoplasmic tail swap experiments that the information for the sub-Golgi concentration of the two glycolipid glycosyltransferases GalNAcT2 and SialT2 is present in their cytoplasmic tail (Uliana et al., 2006). Another study found that several basic amino acids located in the cytoplasmic tail of ST3Gal5, an enzyme involved in ganglioside synthesis, are important in both ER export and Golgi retention (Uemura et al., 2015). Replacement of two arginine residues (R2A/R3A) within the R/K-based motif localised the mutant not only in the Golgi but also in endosomes, resulting in the presence of immature *N*-glycans. This indicated that the R/K-based motif is essential for Golgi retention, and that retrograde transport is necessary for *N*-glycan maturation. The importance of cytoplasmic basic amino acids for ER export has also been demonstrated for a tobacco prolyl hydroxylase and the transcription factor bZIP28, both type II membrane proteins (Yuasa et al., 2005; Srivastava et al., 2012).

Despite the lack of a discernible function in the localization of *N*-glycosylation enzymes in the Golgi, cytoplasmic dihydrophobic and diacidic signal motifs have also been shown to play a role in mediating Golgi localization. For example, hydrophobic residues in combination with a tyrosine residue (FVXXXY) play a role in the ER export of the Arabidopsis multi-TMD protein ENDOMEMBRANE PROTEIN 12 (EMP12; Gao et al., 2012). In yeast, the tyrosine-based motif YYXF found in the cargo receptors Emp47p and Emp46p, which shuttle between the ER and the *cis*-Golgi, is essential for their export from the ER and localization in the Golgi (Schröder et al., 1995; Sato and Nakano, 2002). Similarly, two phenylalanines facilitate the binding of the cargo receptor ERGIC-53, a membrane protein of the ER-Golgi intermediate compartment (ERGIC), to COPII, thereby enabling its ER exit and Golgi localization (Kappeler et al., 1997). In plants, diacidic sequence motifs (D/EXE) have been identified in the N-terminal cytoplasmic tails of type I multispanning membrane protein GONST1 (Golgi nucleotide sugar transporter 1) and the type II Golgi protein CASP (CCAAT-displacement protein alternatively spliced product), both of which rely on diacidic motifs to exit the ER and reach the Golgi (Hanton et al., 2005). However, even after mutating this motif in both fusion proteins, they were still partially localised to the Golgi, suggesting that other factors contributed to their exit from the ER. When an ER-retained synthetic type I reporter protein with a 17-amino acid TMD was fused to a segment of the cytoplasmic region of CASP, it was localised to the Golgi. Mutation of the DXE motif in this reporter protein resulted in its complete retention in the ER, further supporting the role of the DXE motif in ER export. Similarly, ER export and Golgi targeting of the syntaxin of plants (SYP) 3 family protein SYP31, a type II soluble N-ethylmaleimide-sensitive fusion protein attachment protein receptor (SNARE) protein, relies on a diacidic EXXD motif (MELAD) in its N-terminal cytoplasmic tail (Chatre et al., 2009). The potassium channel protein KAT1 requires a diacidic signal for ER exit and subsequent localization to the PM in guard cells (Mikosch et al., 2006). The motif has been shown to interact with SEC24A, a component of the COPII coat (Sieben et al., 2008). This is consistent with a mammalian study showing a preference of the SEC24A isoform for binding to DXE motifs (Chatterjee et al., 2021). SEC24 was also found to bind to an LXX(L/M)E motif in the cytoplasmic N-termini of the two yeast SNAREs Sed5p and Bet1p (Mossessova et al., 2003). The same LXXLE motif was identified in the N-terminus of BET12, the Arabidopsis homologue of Sed5p. This motif in combination with a dibasic motif near the TMD was shown to mediate ER export (Chung et al., 2018).

## ER retention via retrograde transport

Little is known about the presence and involvement of amino acid sequence motifs in the retrieval and retention of *N*-glycan processing enzymes within the Golgi stack. Mammalian Golgi glycosyltransferases and glycosidases were thought to be good examples of retained proteins (Munro, 1991; Nilsson et al., 1991; Machamer, 1993). However, many, if not all, Golgi proteins cycle continuously within the Golgi stack and/or between the Golgi and the ER (Lippincott-Schwartz et al., 2000; Ward and Brandizzi, 2004; Storrie, 2005; Schoberer et al., 2010). This could occur either by regular retrograde transport mediated by COPI-coated vesicles that form at the periphery of Golgi cisternae (Pimpl et al., 2000; Ritzenthaler et al., 2002), a COPI-independent mechanism involving tubules, direct transport to the ER, or a combination of these possibilities (Nichols and Pelham, 1998; Storrie, 2005; Schoberer et al., 2010). Evidence for the cycling of Golgi residents comes, for example, from fluorescence recovery after photobleaching (FRAP) experiments showing the exchange of GFP-labelled Golgi proteins between Golgi and ER pools (Zaal et al., 1999; Miles et al., 2001; Brandizzi et al., 2002a; Schoberer et al., 2010). The predominant means for retrograde transport are COPI vesicles, whose coat is composed of seven individual coat protein subunits, α/β/β’/δ/ϵ/γ/ζ (Pimpl et al., 2000; Ritzenthaler et al., 2002). In plants, two types of COPI vesicles have been identified based on their size. As shown by electron tomography analysis, COPIa vesicles exclusively bud from *cis*-Golgi cisternae and reside at the ER-Golgi interface in Arabidopsis (Donohoe et al., 2007). COPIb vesicles are present at the medial- and *trans*-Golgi cisternae and are most likely responsible for retrograde transport within the Golgi stack.

The best-characterized COPI binding signals include dilysine motifs (KKXX and KXKXX) as described for the p24 protein family and the K/HDEL retrieval signal, which are found on many ER-resident proteins to direct them back to the ER (Contreras et al., 2004; Langhans et al., 2008; Gao et al., 2014). The Arabidopsis glycerol-3-phosphate acyltransferase 8 (GPAT8) and fatty acid desaturase 3 (FAD3) contain C-terminal KK and KXKXX motifs, respectively, that differ slightly from the canonical dilysine motif (McCartney et al., 2004; Gidda et al., 2009). Arginine-based motifs have also been shown to play a crucial role in mediating ER localization. For example, the N-terminal cytoplasmic tail of Arabidopsis GCSI contains four arginine residues that are constitutive for three di-arginine motifs (RR, RXR or RXXR), each of which can confer ER localization most likely through retrieval from the Golgi as mutating all four arginines shifted GCSI localization from the ER to the Golgi (Boulaflous et al., 2008). Fusing the arginine-rich tail of GCSI to the first 35 N-terminal amino acids of XYLT (encompassing the cytoplasmic tail and TMD) redirected the enzyme from the Golgi to the ER. In mammals, the arginine-rich motif RRXXXXR has been demonstrated to retain the GM3 synthase isoform M1-SAT-I, a glycosyltransferase involved in ganglioside synthesis, within the ER. Mutating two of the three arginine residues redirected the enzyme to the Golgi, where its two homologs M2-SAT-I and M3-SAT-I are located (Uemura et al., 2009). ER retention can also be conferred by the hydrophobic pentapeptide motif ϕ-X-X-K/R/D/E-ϕ (where ϕ denotes a hydrophobic amino acid), which is found in the C-termini of Arabidopsis GPAT9 and FAD2 (McCartney et al., 2004; Gidda et al., 2009).

## Golgi retention via retrograde transport

A recent study showed that certain mammalian *cis*-Golgi glycosyltransferases are capable of directly binding to COPI subunits via the specific amino acid motif ϕ-(K/R)-X-L-X-(K/R) in their N-terminal cytoplasmic tails. This interaction is critical for maintaining their correct steady-state location in the *cis*-Golgi (Liu et al., 2018). The same study also showed that δ-COP can bind to the N-terminal tail of GALNT4 via a WX(n1-6)(W/F) motif, which is an evolutionarily conserved δ-COP μ-homology domain (MHD)-interacting motif (Suckling et al., 2015). Notably, most medial and *trans*-Golgi enzymes did not bind to COPI subunits. This suggests that this mechanism may be unique to *cis*-Golgi proteins that require recycling from late Golgi compartments to maintain their steady-state distribution.

Furthermore, glycosyltransferases may interact with COPI via an adaptor-mediated mechanism. In yeast, the peripheral membrane protein Vps74p has been shown to facilitate the sorting and recycling of multiple Golgi-localised glycosyltransferases by binding to a semi-conserved FLS-like motif (F/L-L/I/V-X-X-R/K) present in their cytoplasmic tail and the COPI coat (Tu et al., 2008, 2012). Vps74p thus functions as an adapter linking these enzymes to the COPI machinery, which facilitates the transport of glycosylation enzymes back to earlier Golgi cisternae, thereby enabling their dynamic localization in the Golgi (Schmitz et al., 2008; Tu et al., 2008). For example, the mutation of one or two residues within this motif resulted in the mislocalization of the mannosyltransferase Kre2p to the vacuole (Tu et al., 2008). Golgi phosphoprotein 3 (GOLPH3), the mammalian homologue of Vps74p, has been demonstrated to perform a comparable function (Ali et al., 2012; Eckert et al., 2014; Pereira et al., 2014). Although mammalian glycosyltransferases lack the yeast consensus motif, the two isoforms GOLPH3 and GOLPH3L have been demonstrated to partially compensate for the Vps74p mutant phenotypes (Tu et al., 2008). The amino acid sequence LxxR has been identified in the cytoplasmic tails of potential GOLPH3 binding partners that are involved in glycosphingolipid biosynthesis (Rizzo et al., 2021). For example, GOLPH3 was shown to bind to the N-terminal cytoplasmic tail of the lactosylceramide synthase (LCS) that contains an LPRR motif, which led to its retention at the *trans*-Golgi, where it is sorted into retrograde transport vesicles (Rizzo et al., 2021). Knockdown of GOLPH3 led to its mislocalization to the TGN and then lysosome for its degradation. It has been put forth that TMD- and GOLPH3-dependent sorting occurs primarily in the late Golgi, whereas direct interactions with COPI are observed predominantly in the early Golgi (Welch and Munro, 2019). The human mannosidase ERManI has two di-basic arginine motifs (RRXX) in its N-terminal cytoplasmic tail that are critical for binding to γ-COP, the gamma subunit of COPI (Pan et al., 2013), but has also been proposed to interact with GOLPH3 (Welch et al., 2021). Despite the important function of Vps74p/GOLPH3 in the retrieval and retention of Golgi-resident glycosylation enzymes in yeast and mammals, respectively, no homologues of Vps74p/GOLPH3 have been identified in plants. The only study suggesting adaptor-mediated retrieval of Golgi-resident *N*-glycan processing enzymes in plants is that of the *cis*/medial- enzyme GNTI (Schoberer et al., 2019a). When a conserved polar glutamine (Q) residue in the TMD of AtGNTI or NtGNTI was replaced, the fluorescent fusion protein was mislocalized to the vacuole and *N*-glycan processing was impaired *in vivo*, indicating a TMD-based sorting mechanism. The glutamine residue may facilitate an interaction with an unknown Golgi-resident adaptor protein/complex or may be crucial for a specific protein-lipid interaction to partition GNTI into a specific lipid/membrane domain, which is likely to promote COPI-mediated retrograde transport and thereby maintain the steady-state localization of GNTI in the *cis*/medial Golgi of plants. The newly identified mechanism, which is active in both Arabidopsis and Nicotiana, differs from previous models such as the bilayer thickness model, kin recognition and the more recent cytoplasmic tail-dependent sorting (Tu and Banfield, 2010).

Further support for the involvement of the COPI machinery in the steady-state localization of Golgi-resident glycosylation enzymes comes from the study of the Arabidopsis *cis*-Golgi resident ER-α-mannosidase I (MNS3; Schoberer et al., 2019b). MNS3-GFP was partially translocated to the vacuole when coexpressed with RNAi constructs that silence endogenous δ-COP and ϵ-COP, the delta and epsilon subunits of COPI, in *N. benthamiana* leaves (Schoberer et al., 2019b). These results suggested the involvement of COPI-mediated recycling from *trans*- to *cis*-Golgi cisternae as proposed by the cisternal maturation model, while the trafficking pathway from the Golgi to the vacuole may highlight the default degradation pathway for glycosyltransferases that are no longer needed in a plant cell. A similar shift to the vacuole was shown for GNTI-mRFP upon knockdown of endogenous δ-COP and ϵ-COP (Schoberer et al., 2019a). Although the cytoplasmic tails of MNS3 and GNTI contain numerous basic amino acids that are essential residues of the binding motifs in the cytoplasmic tails of Golgi glycosyltransferases and glycosidases, there is no recognizable canonical COPI binding motif present that could account for the observed mislocalization (**Figure 6A**). This is in contrast to a study of the mammalian MNS3 ortholog, human ERManI, which contains two di-basic arginine motifs (RRXX) required for binding to γ-COP (Pan et al., 2013). Interestingly, the ER localization of the yeast MNS3 ortholog ER α1,2-mannosidase I relies on the interaction of its TMD with retention in endoplasmic reticulum sorting receptor 1 (RER1), a Golgi-localised retrieval receptor that cycles the protein from the Golgi back to the ER (Massaad et al., 1999). Arabidopsis possesses three RER1 family members that can complement the mislocaliation of cargo proteins in the yeast mutant Δ*rer1*. A role for RER1 in intra-Golgi transport or the presence of RER1-interacting proteins has yet to be described in plants.

## Golgi retention via hydrophobic residues

A Golgi retention motif containing an important leucine residue (LPYS) was identified in the cytoplasmic tail of Arabidopsis MNS3 (Schoberer et al., 2019b). Despite its high level of conservation among plant ER-α-mannosidases, this motif does not appear to be related to any of the Golgi localization signals that have been described thus far in yeast, mammals, and plants. When MNS3-GFP was treated with brefeldin A (BFA), a secretory inhibitor, or tagged with the ER-retrieval signal HDEL, which both commonly result in the relocation of Golgi enzymes to the ER, the fluorescent fusion protein remained on dispersed punctate structures resembling Golgi remnants. However, the deletion of the LPYS motif or the exchange of the leucine residue for alanine in the full-length cytoplasmic tail of MNS3 resulted in the relocation of the protein to the ER. This Golgi-to-ER shift may indicate a malfunction in the Golgi retention mechanism, potentially due to the disruption of the binding interaction between the cytoplasmic motif and hitherto unidentified determinants within the Golgi apparatus. Only recently, a conserved di-leucine motif (LXL) near the cytoplasmic C-terminus of the Arabidopsis K/HDEL receptor ERD2 was identified as a crucial determinant of its Golgi residency and biological function, mediating ER retention of soluble ligands (Silva-Alvim et al., 2018). Replacement of the leucine residues resulted in a significant relocation of AtERD2 to the ER. Subsequently, it was demonstrated that the LXL motif functions as a Golgi retention signal, preventing its recycling to the ER, and is required to inhibit COPI-mediated receptor recycling (Alvim et al., 2023).

## Golgi retention via COG-mediated retrograde transport

An important role in the intra-Golgi retrograde trafficking of Golgi-resident *N*-glycan processing enzymes in mammalian and yeast cells has been attributed to the COG complex (Conserved Oligomeric Golgi complex). This multi-protein tethering complex consists of eight subunits, named COG1 to COG8, that are organized into two subcomplexes, lobe A and lobe B. The COG complex is essential for tethering retrograde vesicles within the Golgi, helping to ensure that enzymes and other proteins remain in the correct Golgi compartments and thereby maintaining Golgi homeostasis (Smith and Lupashin, 2008; Blackburn et al., 2019). Live-cell super-resolution microscopy has recently shown that lobe A is preferentially Golgi-bound, whereas lobe B is mainly found on vesicles (Willet et al., 2016). This localization facilitates association between vesicles and target membranes, allowing SNARE complex formation and vesicle fusion. Disruptions in the COG complex can lead to severe diseases known as congenital disorders of glycosylation (COG-CDG), which result from the mislocalisation of *N*-glycosylation enzymes, affecting glycosylation patterns and leading to improperly glycosylated proteins. Although Arabidopsis homologs of all eight COG subunits have been identified in plants (Latijnhouwers et al., 2005), only COG3, COG6, COG7 and COG8 have been functionally studied in Arabidopsis. Studies of *cog3*, *cog6*, *cog7* and *cog8* mutants suggest that the Arabidopsis COG complex plays an important role in maintaining the structural and functional integrity of the Golgi apparatus during pollen tube tip growth (Ishikawa et al., 2008; Tan et al., 2016; Rui et al., 2020). This seems reasonable since pollen tube growth relies on the targeted secretion of vesicles containing cell wall components, such as pectins and hemicelluloses, which are synthesized in the Golgi. Mislocalisation of Golgi-resident enzymes, which are involved in the synthesis of these cell wall polysaccharides, and a defective Golgi stack morphology result in their incorrect deposition, leading to weakened cell walls and impaired pollen tube elongation (Tan et al., 2016). Interestingly, mutations in other proteins involved in vesicle tethering, such as the Golgi-resident Qa-SNARES SYP31 and SYP32 or the RAB GTPases RABD2b and RABD2c, also result in defects in pollen development and pollen tube growth (Peng et al., 2011; Rui et al., 2021). Rui and colleagues recently showed that SYP31 and SYP32 interact with COG6 and are responsible for its Golgi localization in Arabidopsis (Rui et al., 2021).

In summary, the CTS region is essential for the correct localization of *N*-glycosylation enzymes in yeast, mammals and plants (Tu and Banfield, 2010; Schoberer and Strasser, 2011). However, the determinants required for proper Golgi targeting and retention are highly variable, as reflected by the lack of sequence similarity between different glycosylation enzymes (**Figure 6**).

## Significance of *N*-glycan processing enzyme localization in plant glycoengineering

Glycoengineering is the process of manipulating the glycosylation pathways of organisms to create tailored glycoproteins with precise glycan modifications. This is particularly important for the production of glycoproteins used in a therapeutic context, where specific glycan profiles can influence their efficacy, stability, and immunogenicity. For example, protective glycan residues, such as sialic acid, can prolong the half-life of glycoproteins, which is critical for creating stable, long-lasting therapeutic proteins, making them more effective in clinical applications. Plants are an attractive platform for glycoengineering and are increasingly being used as biofactories for the production of therapeutic proteins (Tekoah et al., 2015; Hager et al., 2022; Whaley and Zeitlin, 2022; Zahmanova et al., 2023), because they are scalable, cost-effective, and can be grown in large quantities. However, control of the glycosylation process is essential for the use of plants as commercial platforms for the production of non-plant glycoproteins due to differences in plant-specific glycan structures and their potential incompatibility with human applications. Plants add β1,2-xylose and core α1,3-fucose to complex *N*-glycans, which are absent in humans and potentially immunogenic, limiting the therapeutic use of plant-produced glycoproteins. For example, antibodies used in monoclonal antibody therapy require fucose-depleted glycans to enhance antibody-dependent cell-mediated cytotoxicity, a mechanism that helps the immune system kill target cells. Therefore, glycoengineering efforts have focused on inhibiting the activity of β1,2-xylosyltransferase and core α1,3-fucosyltransferase through knock-down/knock-out strategies, resulting in the generation of plant species that synthesize complex *N*-glycans composed of the GnGn oligosaccharide devoid of plant-specific glycan residues (Castilho and Steinkellner, 2012).

By removing plant-specific glycosylation enzymes, introducing human or mammalian glycosylation enzymes that plants naturally lack, into the appropriate Golgi cisternae and manipulating the localization of resident enzymes within the Golgi stack, scientists can control the sequence of glycan processing, thereby ‘humanizing’ the glycosylation pathways in plants to produce therapeutic proteins that meet regulatory standards for use in humans without the need for costly downstream processing to alter the glycan structures. Knowledge of the critical determinants of Golgi enzyme localization is essential as the mislocalisation of enzymes could result in incomplete or incorrect glycosylation, leading to undesired protein properties. To illustrate, human β1,4-galactosyltransferase (β1,4-GALT), which is absent in plants, is targeted to a distinct Golgi compartment in plants, where it inhibits biantennary complex *N*-glycan formation, resulting in augmented *N*-glycan heterogeneity (Bakker et al., 2001; Strasser et al., 2009). The previous chapters established that the localization of *N*-glycan processing enzymes in the plant Golgi is controlled by the CTS region and that the catalytic domain is responsible for glycan processing activity. Swapping the CTS regions and catalytic domains between enzymes has shown considerable potential for gaining more precise control over glycosylation pathways and for adding additional functions to plant pathways. This is exemplified by the successful generation of branched and galactosylated structures (Castilho et al., 2011; Nagels et al., 2011; Castilho and Steinkellner, 2012). Furthermore, the expression of a chimeric protein comprising the CTS region of ST and the catalytic domain of human β1,4-GALT led to the efficient production of di-galactosylated *N*-glycan structures in *N. benthamiana* (Strasser et al., 2009). In contrast, the CTS region of XYLT fused to the catalytic domain of β1,4-GALT resulted in predominantly mono-galactosylated and hybrid *N*-glycan structures (Bakker et al., 2006). Since galactosylation of *N*-glycans prevents further processing by GMII and GNTII, this incomplete processing of *N*-glycans suggests that the ST-CTS region, in contrast to the XYLT-CTS region, directs targeting to a later Golgi compartment. In addition, the transient expression of the GNTI-CTS region led to a block in *N*-glycan processing on a co-expressed glycoprotein in *N. benthamiana* (Schoberer et al., 2023). The identified sequence motif in the GNTI stem region acts as a dominant-negative sequence motif that can be used in transient glycoengineering approaches for the recombinant production of oligo-mannosidic *N*-glycans.

In addition to mimicking human glycosylation, glycoengineering can create novel glycan structures that do not occur naturally in plants or humans (Kallolimath et al., 2016; König-Beihammer et al., 2021), allowing the synthesis of novel glycoforms with specific biological properties that can be exploited for therapeutic or industrial applications.

## Challenges and future directions

Studying the trafficking of Golgi-resident *N*-glycan processing enzymes in plants is a complex task due to the unique challenges posed by plant cellular architecture and the dynamic nature of the Golgi apparatus. The plant Golgi apparatus is highly dynamic and spatially complex compared to its mammalian and yeast counterparts. It consists of numerous mobile Golgi stacks that move along the actin cytoskeleton, often interacting with the rapidly remodeling ER. Golgi-localised *N*-glycan processing enzymes are also not static, constantly cycling between the ER and the Golgi and within the Golgi. This makes it difficult to capture, track and quantify protein dynamics and determine the specific Golgi compartments where *N*-glycan processing takes place. While mammalian and yeast systems have well-established tools for studying and visualizing protein trafficking, plant-specific tools are still being developed. For example, high-resolution imaging techniques are essential for studying the fine details of enzyme trafficking. However, many fluorescence-based imaging techniques have limitations when applied to plant cells due to the presence of a cell wall that limits the penetration of fluorescent probes, autofluorescence in chlorophyll-containing leaves, a large vacuole that reduces the density of target structures, the thickness of plant tissues, and the rapid movement of organelles. Numerous super-resolution imaging techniques, such as stochastic optical reconstruction microscopy (STORM), photoactivated localization microscopy (PALM) and stimulated emission depletion microscopy (STED) have increased spatial resolution, but are limited at the level of temporal resolution as they necessitate prolonged acquisition times and are therefore incompatible with the imaging of mobile phenomena, such as the rapid remodeling of the ER and movement of Golgi stacks in plants.

A tool that was missing in plants until very recently was the RUSH (Retention Using Selective Hooks) system, which has been used in mammalian cells to study and quantify protein trafficking and dynamics in living cells by allowing researchers to control the retention, release and movement of cargo proteins from a donor compartment within cells in real-time (Boncompain and Perez, 2012). Using the RUSH system in combination with real-time high-resolution imaging, it has been shown that ERES expand into a tubular network connected to the ER, containing secretory cargo but no COPII components. COPII components do not accompany departing cargo containers but instead remain on ERES by decorating the neck of tubules, which form an interwoven network connected to the ER (Westrate et al., 2020; Shomron et al., 2021; Weigel et al., 2021). This suggests that vesicles are not the only means of anterograde transport and is consistent with a recent study performed in *N. tabacum* leaves, which found that ER tubules are often associated with punctate structures where ERES and Golgi markers are co-localised (McGinness et al., 2022). The immobile nature of the mammalian ER and Golgi apparatus is a major advantage when monitoring fluorescent proteins in real time at high resolution. Recently, a version of the RUSH system has been established in plants (Fougère et al., 2025). Utilizing the RUSH system and high-resolution microscopy, the authors have identified a highly dynamic, Golgi-independent *cis*-Golgi tubulo-vesicular network in Arabidopsis, which was proposed to constitute an early station of the ERGIC in plants (**Figure 4**). This study significantly updates previous models of the plant secretory pathway. The existence of a plant ERGIC has been widely debated (Robinson et al., 2015), and this study raises the question of whether the proposed plant ERGIC acts as a new sorting hub for both anterograde and retrograde traffic, as is the case in mammalian cells (Appenzeller-Herzog and Hauri, 2006). This finding certainly introduces additional layers of regulation and complexity to the localization mechanisms of Golgi-resident *N*-glycan processing enzymes by introducing extra steps of sorting, retention, and retrieval steps that could affect the distribution or trafficking dynamics of enzymes. The ERGIC could facilitate more precise spatial and temporal control over the entry of enzymes into the Golgi, potentially impacting the overall accuracy of glycan processing.

The presence of multiple isoforms for glycosylation enzymes and a variety of components of the trafficking machinery, such as RABs, SNAREs, COPI and COPII proteins, adds another layer of complexity in plants. This redundancy makes it difficult to study the function and trafficking of a single enzyme, as knocking out or disrupting one protein of interest may be compensated for by another. This may also explain why knockouts of single proteins in plants are neither lethal nor result in specific phenotypes. The significance of multiple copies for proteins involved in trafficking is unknown but may be related to specificity for a particular type of cargo.

Future research may focus on gaining a mechanistic understanding of the sub-Golgi targeting and protein retrieval and retention mechanisms by studying how specific motifs and domains in *N*-glycan processing enzymes are recognized by the plant Golgi trafficking machinery. To date, little is known about how *N*-glycan processing enzymes are recycled and positioned in the cisternae where they function. The precise role of the COG complex within the plant Golgi is not well understood. Notably, a *cog7* mutant with an amino acid substitution in the conserved domain of the protein displays a highly accelerated dark-induced senescence phenotype (compared to wildtype) that is accompanied by enhanced protein *N*-glycosylation, thereby linking COG function to both *N*-glycosylation and plant stress responses (Choi et al., 2023). Also, unlike in mammals and yeast, plants lack certain sorting factors (e.g. Vps74p/GOLPH3), and the motifs responsible for COPI-mediated retrieval are not conserved. These findings suggest the existence of unique or as-yet-undiscovered plant-specific mechanisms for Golgi enzyme localization and retention.

Similarly, the impact of variations in the lipid environment within Golgi membranes on the localization and function of glycosylation enzymes in plants remains relatively unexplored. Lipids are active participants in the sorting of membrane proteins in the Golgi, rather than passive components. They create specialized membrane environments, regulate vesicle formation, and interact with proteins to ensure the accurate delivery of cargo throughout the cell. Inhibition of glucosylceramide biosynthesis was linked to decreased protein secretion and perturbations of Golgi structure, although the localization of the *trans*-Golgi marker ST remained unaffected (Melser et al., 2010). Only recently, it was reported that the maturation of ERGICs into Golgi cisternae depended on C24-ceramide sphingolipids (Fougere et al., 2025).

Even small changes in the localization of *N*-glycan processing enzymes can lead to abnormal glycosylation, which can have significant effects on protein folding, stability, and function. These changes may not be readily observable, especially in the early stages of mislocalisation, making it difficult to link trafficking defects to physiological effects. Molecular or proteomic studies using mutants with glycosylation and/or trafficking defects will help to elucidate the regulatory mechanisms that control enzyme localization.

## Concluding remarks

The trafficking and precise localization of Golgi-resident *N*-glycan processing enzymes and the precise control of these processes in plants are vital for maintaining proper glycoprotein biosynthesis and function. Understanding these mechanisms is crucial for manipulating glycosylation processes in plants, which can be important for producing glycoproteins for pharmaceutical purposes. Studying the trafficking of Golgi-resident *N*-glycan processing enzymes in plants is challenging due to the dynamic and complex organization of the plant Golgi, the difficulty of distinguishing between distinct compartments within the Golgi stack at the nanoscale and the redundancy of the key components of the protein trafficking machinery. Advanced imaging techniques that combine high-resolution and real-time imaging, tools like the RUSH system, and genetic/proteomic screens of mutants with aberrant glycosylation will provide a deeper understanding of plant-specific trafficking pathways and help overcome these challenges to unravel the precise mechanisms governing the localization and function of glycosylation enzymes in plants, which in turn will expand our understanding of plant cell biology.
